# User experience of instant blood pressure: exploring reasons for the popularity of an inaccurate mobile health app

**DOI:** 10.1038/s41746-018-0039-z

**Published:** 2018-08-10

**Authors:** Timothy B. Plante, Anna C. O’Kelly, Bruno Urrea, Zane T. MacFarlane, Roger S. Blumenthal, Jeanne Charleston, Edgar R. Miller, Lawrence J. Appel, Seth S. Martin

**Affiliations:** 1 Department of Medicine, Larner College of Medicine at the University of Vermont, Burlington, VT USA; 2 Department of Medicine, Massachusetts General Hospital, Boston, MA USA; 3 Division of Cardiology, Department of Medicine, Johns Hopkins University School of Medicine, Baltimore, MD USA; 4 Department of Medicine, MedStar Union Memorial Hospital, Baltimore, MD USA; 5 Department of Chemistry, Pomona College, Claremont, CA USA; 6 Ciccarone Center for the Prevention of Cardiovascular Disease, Division of Cardiology, The Johns Hopkins University School of Medicine, Baltimore, MD USA; 7 Department of Epidemiology, The Johns Hopkins Bloomberg School of Public Health, Baltimore, MD USA; 8 Welch Center for Prevention, Epidemiology, and Clinical Research, The Johns Hopkins University, Baltimore, MD USA; 9 Malone Center for Engineering in Healthcare, Johns Hopkins University Whiting School of Engineering, Baltimore, MD USA

**Keywords:** Epidemiology, Hypertension

## Abstract

Instant blood pressure (IBP) is a top-selling yet inaccurate blood pressure (BP)-measuring app that underreports elevated BP. Its iTunes app store user ratings and reviews were generally positive. Whether underreporting of elevated BP improves user experience is unknown. Participants enrolled at five clinics estimated their BP, measured their BP with IBP, then completed a user experience survey. Participants were grouped based on how their IBP BP measurements compared to their estimated BP (IBP Lower, IBP Similar, or IBP Higher). Logistic regressions compared odds of rating “agree” or “strongly agree” on survey questions by group. Most participants enjoyed using the app. In the adjusted model, IBP Higher had significantly lower proportions reporting enjoyment and motivation to check BP in the future than IBP Similar. All three groups were comparable in perceived accuracy of IBP and most participants perceived it to be accurate. However, user enjoyment and likelihood of future BP monitoring were negatively associated with higher-than-expected reported systolic BP. These data suggest reassuring app results from an inaccurate BP-measuring app may have improved user experience, which may have led to more positive user reviews and greater sales. Systematic underreporting of elevated BPs may have been a contributor to the app’s success. Further studies are needed to confirm whether falsely reassuring output from other mobile health apps improve user experience and drives uptake.

## Introduction

Mobile health (“mHealth”) apps leverage mobile platforms to deliver health and wellness technologies and are sold directly to consumers on commercial app stores. Most US smartphone-owning adults have downloaded ≥1 mHealth app.^1,2^ Consumers consider app ratings in determining which apps to download. For major app stores, ratings are on a five-star scale; higher star ratings indicate a more enjoyable overall user experience. Mean star ratings are strong drivers of app sales as >90% of consumers would be willing to download an app with a mean star rating ≥4 stars, while only 50% and 15% would be willing to download an app with a mean review of three or two stars, respectively.^3^ For mHealth apps, user experience may be at odds with accuracy of medical functionality. For example, a theoretical app that estimates weight using a smartphone’s camera could improve enjoyment and promote a better user experience if it systematically underestimated a user’s weight and presented reassuring, lower-than-expected weight measurements to users. Such an app could foster greater app use and receive positive reviews despite its inaccuracy, thereby driving uptake of this app over other, more accurate apps. However, if users perceived this app as inaccurate, they may be less likely to use it.

Instant blood pressure (IBP; AuraLife, Newport Beach, CA) is an inaccurate blood pressure (BP)-measuring app that was available on the iTunes app store from June 4, 2014 through July 30, 2015.^4–6^ It was removed from iTunes for unclear reasons after a series of successes. The app was celebrated in the mobile development community, receiving venture capitalist funding from K5 Ventures and winning a competitive Innovation Fund SoCal award, which provides funding to innovation-led startups.^7,8^ User uptake of the app was high as it was downloaded >148,000 times and earned >$600,000. It was in the top 50 most-downloaded apps on all of iTunes for 156 days out of the 421 days that it was available, outranking the for-sale version of the popular video game Angry Birds on 72 of these days.^9^ IBP was highly rated, with a 4.0 mean star rating for the most recent version of the app.^10^

The method by which IBP produces a measurement has not been revealed by the manufacturers, but it involves placing the index finger on the illuminated flashlight and camera while holding the phone’s microphone against the chest. While many of the company’s resources describing app use have been modified or deleted since its removal from iTunes, many details on app use are archived online.^11–17^ We previously published the results of an independent, investigator-initiated validation study of IBP that compared the app to a validated, automated, research-grade oscillometric BP monitor in the Summer and Fall of 2015.^6^ This protocol was based on well-established guidelines for validation of BP-measuring devices. We found the mean (standard deviation (SD)) absolute difference between IBP and the standard device to be 12.4 (10.5) mm Hg for systolic BP and 10.1 (8.1) mm Hg for diastolic BP. IBP misclassified 78% of hypertensive range (≥140/90 mm Hg) BPs as nonhypertensive and achieve the lowest possible British Hypertension Society accuracy grade.

Following publication of our validation study, the Federal Trade Commission pursued litigation against AuraLife, citing false or unsubstantiated claims about the measurement of blood pressure, deceptive use of endorsements, and failure to disclose material connections.^4,18^ AuraLife eventually settled for approximately $600,000, with payment suspended for lack of funds.

Given the high popularity and lack of promised functionality, IBP is a prominent example of a “snake oil” app^19,20^ that exposes its users to potential harm through poor functionality. Understanding the reasons for the high popularity of this inaccurate app will help inform future policy development to ensure a safe app space.

We hypothesized that the systematic underreporting of elevated BPs improved user experience. Specifically, we hypothesized that lower-than-expected IBP-reported BP was associated with a more positive user experience than similar-to-expected or higher-than-expected IBP-reported BP. This would be classified by higher reported app enjoyment and greater likelihood of wanting to check BP in the future. We hypothesized that user-perceived accuracy of IBP would be higher for similar-to-expected BP than those with lower- and higher-than-expected BP.

## Results

### Eligibility

Between August 7, 2015 and September 14, 2015, we obtained informed consent from 101 participants, 3 of whom were excluded due to unavailable cuff sizes or standard device errors, and 17 excluded due to missing IBP values. As shown in Supplementary Table 1, adults excluded for missing measurements were more often female, had lower proportion of hypertension, and were less likely to be of Hispanic ethnicity.

**Fig. 1:**
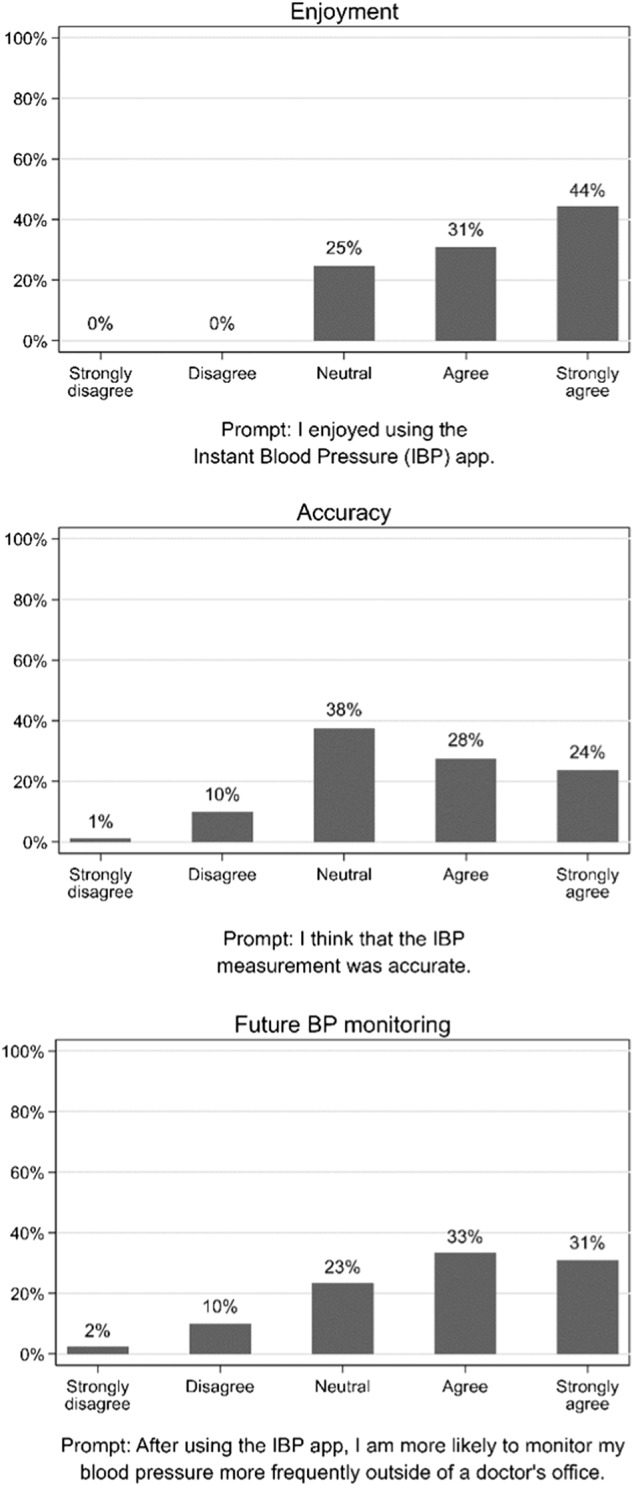
Distribution of survey responses and survey prompts

### Baseline characteristics

Among the 81 individuals included in the sample, the mean (SD) age was 57 (16) years, 54% were men, and 84% had a smartphone (Table 1). The mean of the participant’s estimation of their systolic BP was 126 (15) mm Hg. The first IBP systolic BP was 125 (12) mm Hg. Among participants in this sample, 25 were included in the IBP Lower group, 34 were in the IBP Similar group, and 22 were in the IBP Higher group. Mean age and BMI were similar between groups. The IBP Lower group had a smaller proportion of men than the other groups. The IBP Similar group had greater smartphone ownership than the other groups and the lowest proportion of college-educated participants. The mean (SD) relative difference between the first IBP measurements and the participant’s self-estimation of their systolic BP were −18 (6), 0 (6), and +18 (7) mm Hg for the IBP Lower, IBP Similar, and IBP Higher groups, respectively.

**Table 1:** Baseline demographics of the 81 individuals in the user experience analysis^a^

					*P* comparing groups		
	All participants	IBP Lower	IBP Similar	IBP Higher	Lower vs. similar	Similar vs. higher	Lower vs. higher
N	81	25	34	22			
Age, y	57 (16.4)	54 (16.4)	57 (17.8)	62 (13.4)	0.45	0.26	0.06
BMI, kg/m^2^	27 (6.4)	26 (6.2)	28 (6.8)	28 (6.3)	0.50	0.72	0.33
Male sex, %	54	44	59	59	0.03	>0.99	0.03
Has a smartphone, %	84	80	91	77	0.03	0.01	0.61
Has an mHealth app, %	44	60	39	35	0.13	0.81	0.13
Hypertension, %	57	60	50	64	0.16	0.05	0.56
On an antihypertensive, %	91	87	88	100	0.89	0.18	0.16
Measures BP monthly or more outside of the doctors office, %	52	52	53	50	0.89	0.67	0.78
White race, %	63	60	71	55	0.10	0.02	0.47
Hispanic ethnicity, %	5	4	9	0	0.15	<0.01	0.04
College education, %	81	88	71	91	< 0.01	<0.001	0.49
*Participant’s self-estimation of their own BP*							
Systolic, mm Hg	126 (15)	138 (12)	126 (11)	114 (14)	< 0.001	<0.01	<0.001
Diastolic, mm Hg	74 (10)	76 (11)	76 (9)	69 (9)	0.92	0.01	0.03
IBP BP measurement							
Systolic, mm Hg	125 (12)	120 (11)	125 (10)	132 (13)	0.06	0.04	<0.01
Diastolic, mm Hg	77 (6)	76 (6)	78 (6)	78 (6)	0.25	0.77	0.19
*Difference, IBP BP measurement minus self-estimation*							
Systolic, mm Hg	−1 (15)	−18 (6)	0 (6)	18 (7)	<0.001	<0.001	<0.001
Diastolic, mm Hg	3 (9)	0 (11)	2 (8)	9 (7)	0.542	0.001	0.002

^a^Presented as mean (SD) for continuous variables and proportions for dichotomous variables. IBP Lower had IBP systolic BP > 10 mm Hg below the participant’s self-estimation of their own BP, IBP Similar had IBP systolic BP within 10 mm Hg of the participant’s self-estimation, and IBP Higher had IBP systolic BP > 10 mm Hg above the participant’s self-estimation. Proportions are compared with *χ*^2^ and continuous variables are compared with two-tailed *t* tests.

### User experience survey

Prompts from the user survey are presented in Fig. 1. Among the 81 participants included in the user experience analysis, the majority of participants agreed or strongly agreed with statements affirming that they enjoyed using IBP (75%), that they perceived IBP to be accurate (52%), and that they had an increased likelihood of future BP measurement after using IBP (64%).

### User experience by group

**Fig. 2:**
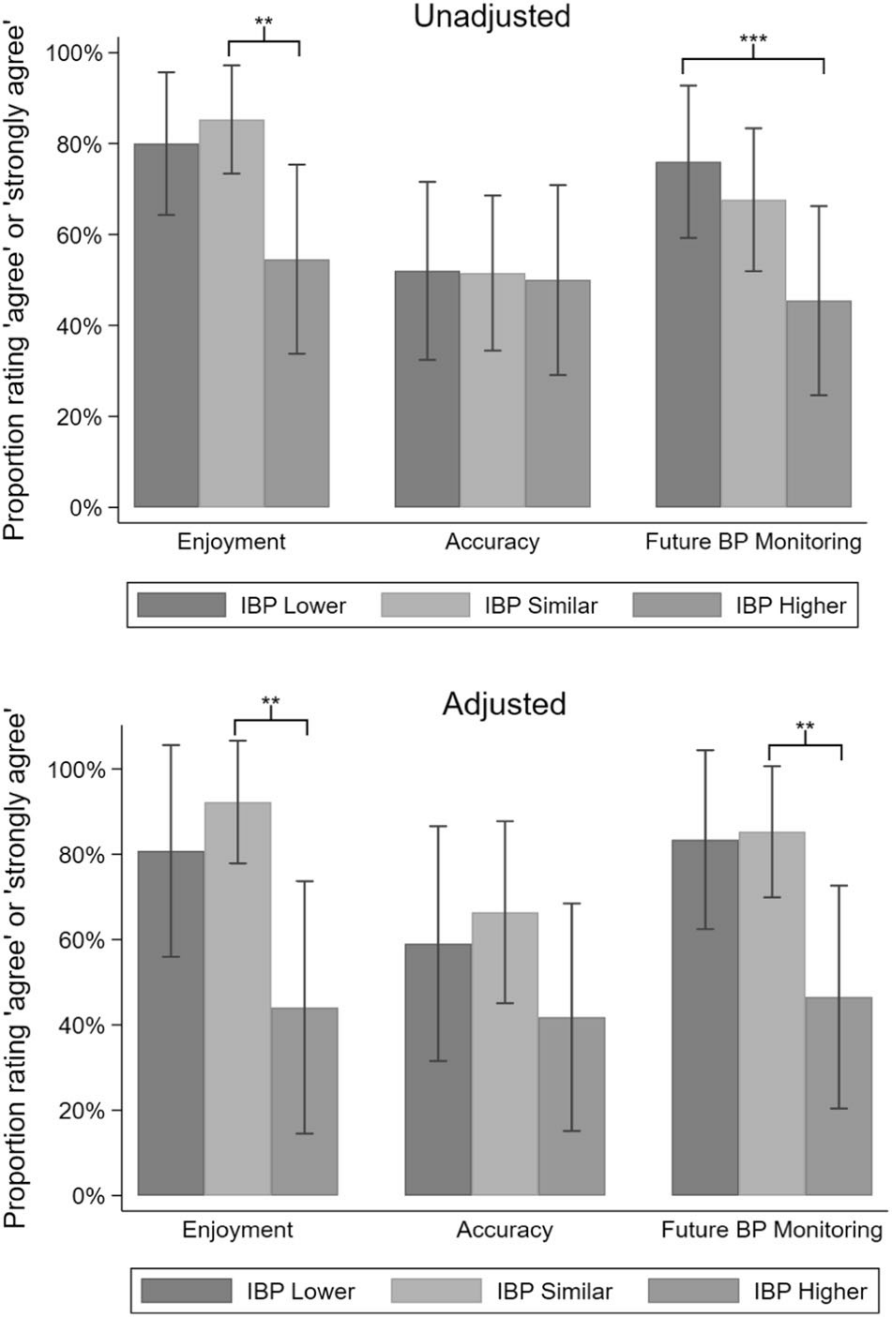
Proportion rating “agree” or “strongly agree” for each domain in the survey, unadjusted and adjusted models*. *The bars indicate a 95% confidence interval surrounding the point estimate of proportions. The adjusted model accounts for age, sex, race, level of education, history of hypertension, receipt of antihypertensives, smartphone ownership, and history of mHealth app ownership. Proportions were converted from odds and were not bounded to 100%. Two stars indicates that the IBP Similar group is significantly different from the IBP Higher group. Three stars indicates that the IBP Lower group is significantly different from the IBP Higher group

In the unadjusted model, the IBP Lower and IBP Similar groups had approximately equal proportions of participants who agreed or strongly agreed with each domain (Fig. 2). IBP Lower, IBP Similar, and IBP Higher groups had comparable numbers of participants reporting that they perceived IBP to be accurate. When compared to the IBP Similar group, IBP Higher had a significantly smaller proportion of participants who reported enjoying using IBP (*P* = 0.015). This domain showed a nonsignificant trend (*P* = 0.067) when comparing the IBP Lower vs. Higher groups. When compared to IBP Higher, IBP Lower had significantly more participants who reported that after using IBP, they were more likely to monitor their BP in the future (*P* = 0.035). The proportion for the IBP Similar group did not differ significantly from the proportion of the IBP Higher group for this domain (*P* = 0.103).

After adjusting for the independent variables, the IBP Lower and IBP Similar groups still had comparable proportions of participants who agreed or strongly agreed with each domain. Likewise, there were no differences between the proportion of participants in any of the three groups regarding perceived IBP accuracy. As in the unadjusted model, there was a significantly smaller proportion of participants who reported enjoying using IBP in the IBP Higher group than in the IBP Similar group (*P* = 0.028). This comparison was not true for the IBP Higher group and the IBP Lower group (*P* = 0.143). In comparison to the IBP Higher group, there was a significantly greater proportion of participants in the IBP Similar group who reported that after using IBP, they were more likely to monitor BP in the future (*P* = 0.036). There was a nonsignificant trend for lower proportion in the IBP Lower group than the IBP Higher group for this domain (*P* = 0.071).

## Discussion

This analysis of user experience of participants enrolled in a validation study of a popular, but inaccurate BP-measuring mHealth app yielded three key findings. First, perceived accuracy was similar regardless of whether the app reported systolic BP that was lower-than-, similar-to-, or higher-than-user-expected systolic BP. Second, there was a negative association between reported enjoyment of the app and higher-than-expected, but not lower-than-expected, systolic BP results. Third, there was a negative association between reported desire to measure BP in the future and higher-than-expected, but not lower-than-expected systolic BP results.

Previous medical literature has explored the usability of mHealth apps developed by medical professionals.^21–24^ To our knowledge, our study is the first to explore user perception of a popular commercial mHealth app. Our findings are an initial step to understanding the role of user experience in the uptake of mHealth apps. For BP-measuring apps, the primary driver of use from a medical perspective should be accuracy. However, our exploration of user experience suggests that falsely reassuring BP results may play a role in promoting app use. In other words, inaccurate apps like IBP may be preferentially used because of a systematic underreporting of elevated BP results. Absent of formal validation, the app store ecosystem may drive use of inaccurate apps. These findings may be explained by a common cognitive process known as the self-serving bias.

Self-serving bias is a common self-protection strategy in which individuals take credit for personal successes but deflect blame for personal failures to external factors.^25–27^ A patient with hypertension, for instance, may blame a high BP reading on heavy traffic during their commute but congratulate themselves on their healthy lifestyle after seeing a reassuring BP reading. As predicted by the self-serving bias, participants whose BP readings by IBP were lower-than-expected or similar-to-expected should be pleased with the results of the app, while participants who receive higher-than-expected BPs may project blame onto IBP and, therefore, have a less positive user experience. IBP’s underreporting of elevated BP measurements may have driven positive user reviews and sales.

User experience represents a complex relationship between a person and a technology. Walter’s Hierarchy of User Needs is a conceptual model striving to define user experience by level of fulfillment that is based on Maslow’s Hierarchy of Needs.^28^ In order to optimize user experience, design must meet basic user needs like functionality (most fundamental), reliability, and usability before promoting enjoyment (most advanced). We can leverage this framework to understand why enjoyment was lower in the IBP Higher but not IBP Lower group. We analyzed participants who experienced similar app functionality by excluding those who did not receive an IBP BP measurement. We have no reason to conclude that usability differed between groups in this analysis. Thus, perceived reliability, or in epidemiological terms, precision, is the variable that differed between groups. Both IBP Lower and IBP Higher groups received IBP systolic BP results that were a similar distance from what they estimated. However, IBP Higher may have perceived poorer reliability, adversely affecting app enjoyment.

Noninvasive BP monitors are class II (moderate-risk) Food and Drug Administration devices that must be validated prior to their release.^29^ The risk with failing to validate these devices, as we have shown with the IBP app, is that patients without access to a validated BP monitor may rely on them to generate inaccurate data and perceptions about their health. These findings underscore the need to validate high-risk mHealth apps prior to their commercial availability, as well as educate patients and providers about the potential perils of these apps. There are several limitations in this analysis. We enrolled a convenience sample of ambulatory patients, not all of whom were smartphone owners. Our sample is therefore imperfect in representing all potential IBP owners. We used a survey that has not been validated to assess user impressions. It is unclear if prolonged use of such an app would alter the user experience, especially among those with access to a validated BP device. We did not assess the impact of variation in the diastolic BP result. We did not assess confidence in one’s self-estimation of their BP. Finally, we did not formally ask if study participants had heard of IBP or had used it previously. Anecdotally, none of those performing data collection (T.B.P., B.U., and Z.T.M.) had a study participant comment that they were familiar with the app.

In conclusion, mHealth app popularity does not necessarily correlate with app accuracy. Furthermore, user experience may be driven more by reassuring results than by app accuracy. This may lead to preferential use of inaccurate apps providing reassuring results over accurate, medically regulated and approved devices. Future research should confirm these findings in a broad assortment of mHealth apps.

## Methods

### Participants

**Fig. 3:**
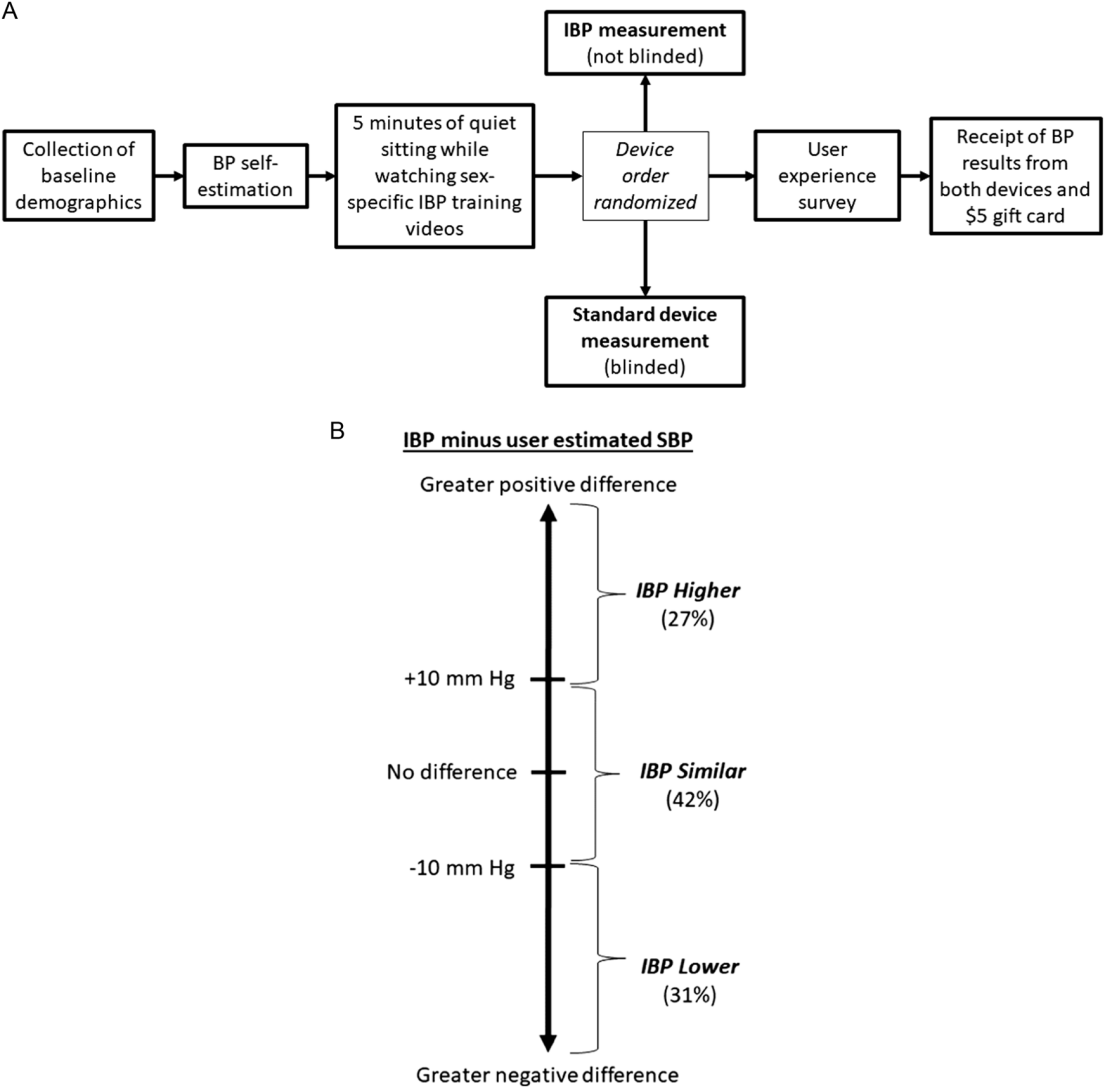
Study flow of the IBP validation study and visualization of group definitions*. *Panel A depicts the flow of the complete IBP validation study. The present analysis was a preplanned substudy embedded within the IBP validation study. Results from the standard device measurement was not incorporated into the present analysis as participants were blinded to the results until they completed the user experience survey. Panel B visually depicts the group definitions and percentage of all in each group

The present user experience study was a prespecified substudy within the IBP validation study. Many details of the IBP validation study have been previously published.^4^ Overview of the flow of the validation study is shown in Fig. 3a. In August and September 2015, we recruited adults with or without hypertension from four Johns Hopkins ambulatory clinics and an ambulatory research clinic. Participants were referred by their physicians or clinical research staff.

The research was conducted in accordance with relevant guidelines except for choice modifications to account for participant movement with IBP measurement, described elsewhere.^5,6,30,31^ This protocol was approved by a Johns Hopkins University Institutional Review Board. All participants provided full written, informed consent.

### Eligibility

Inclusion criteria included age ≥18 years and reported height and weight within IBP’s supported range (3′ 6″–7′ 0″ and 65–450 lbs). Exclusion criteria included contraindication for BP measurement with an arm cuff in both arms, unavailable cuff sizes to match arm circumference, pregnancy, or standard device errors, as well as active arrhythmia or implantable devices. To limit influences from app malfunction on user experience, this analysis excludes all participants in whom IBP did not report a measurement.

### Baseline data

At enrollment, participants reported date of birth, sex, height, weight, race, ethnicity, and highest level of education. Participants reported a history of hypertension, receipt of hypertension medications, home BP monitoring frequency, use of mobile devices, and use of mHealth apps. Participants self-estimated their current systolic and diastolic BP prior to obtaining any study measurements. Participants were asked by study staff to give a specific numerical estimate for each number (e.g., “130 over 80”), rather than subjective interpretation (e.g., “It’ll be high”) or a range (e.g., “Between 130–140 and 80–90”).

### BP measurement protocol

Before any BP measurements were obtained, participants viewed all available manufacturer-produced sex-specific informational videos on a portable video player.^13–17^ Following 5 min of quiet sitting, a pair of BP readings were taken from either IBP or a standard sphygmomanometer.^32^ The order of device pairs (IBP measurement pair first or standard device measurement pair first) was randomized. As participants were their own observers, they were not blinded to IBP measurements. Using the app required participants to view the screen and follow app instructions throughout the measurement process. We did not blind the participants to the results as it would have required the users to close their eyes and not view the on-screen IBP instructions, which is against manufacturer advice. Participants were blinded to the standard device measurement until after the completion of a survey. As the participants were blinded to the standard device measurements until completion of the survey, the BP measurements from the standard devices are considered extraneous to the present analysis and are reported in the main IBP validation study manuscript.

IBP measurements were obtained from IBP version 1.2.3 (AuraLife, Newport Beach, CA) on an iPhone 5s and iPhone 6 (Apple Inc, Cupertino, CA). Study team members were trained to follow a protocol using manufacturer instructions. After obtaining measurements from IBP and the standard device and completion of the user experience survey, participants received a $5 gift card and a written copy of the measurements from both devices.

### User experience survey

We assumed that IBP users do not measure their BP with a sphygmomanometer and the IBP device and that their estimated BP was thus subjective. Further, we suspect that users willing to purchase this $4.99 app are motivated to monitor their BP and that user experience would be adversely affected by an IBP measurement higher than what they had expected to receive. Therefore, we predicted that a user’s initial impression of the app was a function of a user’s expected BP and how this expected value compared to the first measurement they received after using the IBP device. To assess this, participants completed a survey immediately after collection of BP measurements but before learning the results from the reference BP measurement device. These included questions on (1) perceived accuracy of IBP, (2) user enjoyment of IBP, and (3) likelihood of future BP self-monitoring. Each question was scored on a five-point Likert scale (strongly disagree, disagree, neutral, agree, strongly agree).

### Analysis

We sought to understand the association of higher-than, similar-to, and lower-than-expected IBP results with survey responses. As elevations in systolic BP are disproportionately associated with higher risk for cardiovascular disease than are elevations in diastolic BP,^33^ clinical evidence is much stronger for pharmacologic systolic BP-targeting therapy than diastolic BP-targeting therapy,^34,35^ and IBP supports a wider range of systolic BP (102–158 mm Hg or a 57 mm Hg range) than diastolic BP range (65–99 mm Hg or a 35 mm Hg range), we focused analysis on systolic BP. Finally, as IBP had low intra-measurement variability,^36^ we included only the first IBP measurement.

The exposure of interest was the difference between IBP-reported systolic BP and participant-estimated systolic BP. Participants were classified as being in one of three groups (Fig. 3b), IBP Lower (IBP-reported systolic BP ≥ 10 mm Hg lower than participant-estimated systolic BP), IBP Similar (IBP-reported systolic BP within 10 mm Hg of participant-estimated systolic BP), or IBP Higher (IBP-reported systolic BP ≥ 10 mm Hg higher than participant-estimated systolic BP). The outcome of interest was the proportion of each group scoring ≥4 (agree or strongly agree) for each of the questions on the survey. For all participants, the responses for each domain in the survey were tabulated. The percentage of responses for each of the five Likert options was displayed graphically.

We used logistic regression to calculate odds, which we converted to proportions. We used two models, the first was unadjusted. The second was adjusted for age, sex, race, level of education, history of hypertension, receipt of antihypertensives, smartphone ownership, and history of mHealth app ownership.

### Data availability

Scientists wishing to use the IBP validation study data for noncommercial purposes can obtain a deidentified dataset by contacting Dr. Plante.

## Electronic supplementary material

Supplementary Table 1

## Electronic supplementary material

**Supplementary information** accompanies the paper on the *npj Digital Medicine* website (10.1038/s41746-018-0039-z).

## References

[1] Aitken M. and Lyle J. *Patient adoption of mHealth: Use, Evidence and Remaining Barriers to Mainstream Acceptance*. (IMS Institute for Healthcare Informatics, 2015).

[2] Krebs P, Duncan DT (2015). Health app use among US mobile phone owners: A National Survey. JMIR MHealth UHealth.

[3] Walz, A. & Ganguly, R. *The Mobile Marketer’s Guide to App Store Ratings and Reviews, 2015 edition*. (Apptentive).

[4] Marketers of Blood Pressure App Settle FTC Charges Regarding Accuracy of App Readings | Federal Trade Commission. Available at: http://www.webcitation.org/6zRGqDyap. (Accessed 2017).

[5] Plante TB, Appel LJ, Martin SS (2016). Critical Flaws in the Validation of the Instant Blood Pressure Smartphone App-A Letter from the App Developers-Reply. JAMA Intern Med.

[6] Plante TB (2016). Validation of the instant blood pressure smartphone app. JAMA Intern. Med..

[7] Innovate!SoCal 2015—Agenda. Available at: http://innovatesocal.com/agenda.php. Accessed 2018.

[8] Portfolio. *K5 Ventures* Available at: http://k5ventures.com/portfolio/. Accessed: 2018.

[9] App Analytics by Appfigures. *Appfigures*. Available at: https://appfigures.com/reports/top-apps. Accessed 2018.

[10] Plante, T. B. et al. Trends in user ratings and reviews of a popular yet inaccurate blood pressure-measuring smartphone app. *J. Am. Med. Inform. Assoc.*10.1093/jamia/ocy060 (2018). 10.1093/jamia/ocy060 PMC6664848 29878236

[11] The March 29, 2015 version of the Instant Blood Pressure website on Archive.org (2015).

[12] The March 29, 2015 version of the Instant Blood Pressure iTunes App Store listing on Archive.org. Available at: https://web.archive.org/web/20150329054035/https:/itunes.apple.com/us/app/instant-blood-pressure-monitor/id835333129?mt=8 (2015) Accessed 2018.

[13] Female Basic Training Video. Available at: https://vimeo.com/125312429 (2015) Accessed 2015.

[14] Female Advanced Training Video—Clothing Placement. Available at: https://vimeo.com/125526787 (2015) Accessed 2015.

[15] Female Advanced Training Video—Under the Breast Placement. Available at: https://vimeo.com/125331417 (2015) Accessed 2015.

[16] Male Basic Training Video. Available at: https://vimeo.com/127681143 (2015) Accessed 2015.

[17] Male Advanced Training Video. Available at: https://vimeo.com/127683560 (2015) Accessed 2015.

[18] Federal Trade Commission vs. Aura Labs Inc and Ryan Archdeacon.

[19] Steinhubl SR, Topol EJ (2018). Digital medicine, on its way to being just plain medicine. Npj Digit. Med..

[20] “Snake Oil” medical apps continue to be a problem. *iMedicalApps* (2017).

[21] Ding, H. et al. User experience of an innovative mobile health program to assist in insulin dose adjustment: outcomes of a proof-of-concept trial. *Telemed. J. E-Health Off. J. Am. Telemed. Assoc*. 10.1089/tmj.2017.0190 (2017). 10.1089/tmj.2017.0190 29261476

[22] Holtz, B. E. et al. The design and development of MyT1DHero: A mobile app for adolescents with type 1 diabetes and their parents. *J. Telemed. Telecare*. 10.1177/1357633X17745470 (2017). 10.1177/1357633X17745470 29228854

[23] Langius-Eklöf A (2017). Adherence to report and patient perception of an interactive app for managing symptoms during radiotherapy for prostate cancer: descriptive study of logged and interview data. JMIR Cancer.

[24] Desteghe L (2017). The health buddies app as a novel tool to improve adherence and knowledge in atrial fibrillation patients: a pilot study. JMIR MHealth UHealth.

[25] Campbell WK, Sedikides C (1999). Self-threat magnifies the self-serving bias: a meta-analytic integration. Rev. Gen. Psychol..

[26] Easton JF, Stephens CR, Román Sicilia H (2017). The effect of a medical opinion on self-perceptions of weight for Mexican adults: perception of change and cognitive biases. BMC Obes..

[27] Vaillancourt T (2013). Students aggress against professors in reaction to receiving poor grades: an effect moderated by student narcissism and self-esteem. Aggress. Behav..

[28] Walter, A. *Designing for Emotion*. (A Book Apart, 2011).

[29] Ho, C. *Noninvasive Blood Pressure (NIBP) Monitor Guidance*. (1997).

[30] Graves, J. W. & Quinn, D. *Noninvasive Sphygmomanometers—Part 2: Clinical Investigation of Automated Measurement Typ*e. 81060–2, (ANSI/AAMI/ISO, 2013).

[31] Appel LJ, Whelton PK, Seidler AJ, Patel AR, Klag MJ (1990). The accuracy and precision of the Accutracker ambulatory blood pressure monitor. Am. J. Epidemiol..

[32] El Assaad MA, Topouchian JA, Darne BM, Asmar RG (2002). Validation of the Omron HEM-907 device for blood pressure measurement. Blood Press. Monit..

[33] Strandberg TE, Pitkala K (2003). What is the most important component of blood pressure: systolic, diastolic or pulse pressure?. Curr. Opin. Nephrol. Hypertens..

[34] Whelton, P. K. et al. 2017 ACC/AHA/AAPA/ABC/ACPM/AGS/APhA/ASH/ASPC/NMA/PCNA Guideline for the prevention, detection, evaluation, and management of high blood pressure in adults: executive summary: a report of the American College of Cardiology/American Heart Association Task Force on Clinical Practice Guidelines. *Hypertension*. 10.1161/HYP.0000000000000066 (2017).

[35] Qaseem A (2017). Pharmacologic treatment of hypertension in adults aged 60 years or older to higher versus lower blood pressure targets: a clinical practice Guideline From the American College of Physicians and the American Academy of Family Physicians. Ann. Intern. Med..

[36] Plante, T. B. *Validation of the Instant Blood Pressure App*. (2016). 10.1001/jamainternmed.2016.0157 PMC4922794 26938174

