# Supplementary material for: User experience of instant blood pressure: exploring reasons for the popularity of an inaccurate mobile health app
Source: NPJ Digit Med. 2018 Aug 10;1:31. doi: 10.1038/s41746-018-0039-z (PMC6550164; doi:10.1038/s41746-018-0039-z)
Supplement: Supplementary file 1 — Supplementary Table 1 [file 41746_2018_39_MOESM1_ESM.docx]

Supplementary Table 1 - Baseline demographics among all enrolled in the IBP validation trial by missingness of IBP measurements*

|  | No missing IBP measurements | Missing IBP measurements | P-value |
| --- | --- | --- | --- |
| **N** | 81 | 17 |  |
| **Age, y** | 57 (16.4) | 60 (13.7) | 0.52 |
| **BMI, kg/m^2^** | 27 (6.4) | 30 (5.3) | 0.069 |
| **Male sex, %** | 54 | 24 | <0.001 |
| **Has a smartphone, %** | 84 | 88 | 0.42 |
| **Has an mHealth app, %** | 44 | 12 | 0.21 |
| **Hypertension, %** | 57 | 41 | 0.02 |
| **On an antihypertensive, %** | 91 | 85 | 0.64 |
| **Measures BP monthly or more outside of the doctor’s office, %** | 52 | 35 | 0.02 |
| **White race, %** | 63 | 53 | 0.15 |
| **Hispanic ethnicity, %** | 5 | 0 | 0.02 |
| **College education, %** | 81 | 53 | <0.001 |
| **Participant’s self-estimation of their own BP** | | | |
| **Systolic, mm Hg** | 126 (15) | 124.8 (12.5) | 0.74 |
| **Diastolic, mm Hg** | 74 (10) | 74.4 (9.0) | 0.84 |
| **IBP BP measurement** | | | |
| **Systolic, mm Hg** | 125 (12) | 131.3 (2.2) | 0.33 |
| **Diastolic, mm Hg** | 77 (6) | 75.0 (0.8) | 0.43 |
| **Difference, IBP BP measurement minus self-estimation** | | | |
| **Systolic, mm Hg** | -1 (15) | 3 (17) | 0.58 |
| **Diastolic, mm Hg** | 3 (9) | -1 (11) | 0.35 |

*Only those without missing IBP measurements were included in the present study. Presented as mean (SD) for continuous variables and proportions for dichotomous variables. IBP Lower had IBP systolic BP >10 mm Hg below the participant’s self-estimation of their own BP, IBP Similar had IBP systolic BP within 10 mm Hg of the participant’s self-estimation, and IBP Higher had IBP systolic BP >10 mm Hg above the participant’s self-estimation. Proportions are compared with χ^2^ and continuous variables are compared with two-tailed T-tests.
